# Effect of Personalization in Children's Fables

**DOI:** 10.12688/f1000research.177575.1

**Published:** 2026-03-19

**Authors:** Filippo Aschieri, Matilde Cappelletti, Luisa Cassera, Arianna Consentino, Giulia Martellucci, Fabrizio Gervasoni, Aurora Milesi

**Affiliations:** 1 European Center for Therapeutic Assessment, Milan, Italy, 20123, Italy; 2 Department pf Psychology, Università Cattolica del Sacro Cuore, Milan, Italy, 20123, Italy; 3 Home Care, ASST Fatebenefratelli Sacco, Milan, Italy; 4 Industrial Engineering PhD Program, Industrial Engineering Technologies for Sports Medicine and Rehabilitation, University of Rome Tor Vergata, Rome, Italy

**Keywords:** Psychological Assessment, Therapeutic Assessment, Family Assessment, Child Assessment.

## Abstract

Therapeutic Assessment of families with children emphasize children’s active involvement and the use of developmentally appropriate feedback formats. Narrative and metaphor-based approaches, such as therapeutic fables, have been proposed as effective tools to communicate assessment-related information in a way that is emotionally accessible and engaging for children. Personalization is assumed to be a key mechanism underlying their effectiveness; however, little is known about whether increasing levels of personalization yield incremental benefits.

The present study examined the impact of different degrees of personalization in a therapeutic fable on children’s appreciation of the narrative. A total of 222 children aged 9 to10 years were randomly assigned to one of four experimental conditions: non-personalized, minimally personalized, moderately personalized, or fully personalized fable. Following story exposure, children completed the Fable Appreciation Questionnaire assessing affective engagement and perceived narrative quality.

Results indicated that personalized fable elicited significantly higher affective engagement compared to non-personalized narratives, whereas no differences emerged in other formal qualities. Importantly, minimal personalization was sufficient to enhance emotional engagement, with no additional gains observed for higher levels of personalization.

These findings suggest that personalization primarily influences children’s emotional involvement rather than their evaluation of narrative structure, supporting the use of even minimally personalized narratives as an efficient and developmentally sensitive feedback tool.

## Introduction

Within contemporary models of psychological assessment, increasing emphasis has been placed on the active involvement of clients, with assessment increasingly conceptualized as a collaborative and meaning-making process rather than a unidirectional diagnostic procedure. ^1^ This perspective is central to the Therapeutic Assessment (TA ^1– 3^) framework, which views assessment itself as a potential intervention fostering insight and emotional engagement through personalized feedback. ^4, 5^

When assessment involves children, active involvement becomes particularly delicate. Traditionally, feedback has been addressed primarily to parents, with children as passive participants of the assessment process. However, children are increasingly recognized as active participants and legitimate recipients of feedback about their own experiences, emotions, and behaviors, ^6, 7^ requiring developmentally appropriate formats that allow them to understand and emotionally process assessment findings. ^8^

Narrative and metaphor-based approaches have been proposed as especially suitable for this purpose. Therapeutic stories offer a symbolic and psychologically safe space in which children can recognize themselves through characters and plots that mirror their experiences. ^9^ Empirical research shows that narrative exposure supports emotional understanding, empathy, and broader socio-emotional functioning in childhood. ^10^

Preliminary evidence suggests that personalization may be a key active ingredient of therapeutic stories, enhancing engagement by increasing self-relevance. ^11^ However, it still remains unclear whether increasing degrees of personalization can produce incremental benefits. In light of these considerations, the present study aimed to examine whether varying levels of personalization in a therapeutic story influence children’s emotional engagement with the narrative and their perceptions of its overall quality. The study was guided by two primary hypotheses: (1) that increasing levels of personalization would be associated with higher affective engagement with the story, and (2) that personalization would not influence the perceived cognitive complexity or formal qualities of the narrative.

## Methods

### Sample

The sample included a total of 222 children attending the fourth or fifth grade of primary school, recruited from public educational institutions in Northern and Central Italy, of which 97 were females (43.7%) and 125 were males (56.3%). Participants ranged in age from 9 to 10 years, with a mean age of 9.31 years (SD = 0.46); specifically, 154 children (69.4%) were 9 years old and 68 children (30.6%) were 10 years old. With respect to geographical distribution, 110 participants (49.5%) were recruited from schools located in central Italy, whereas 112 participants (50.5%) attended schools in northern Italy.

Since reading comprehension represented an essential skill to read narrative material, inclusion criteria included the absence of known cognitive or linguistic impairments.

Participation was voluntary, and written informed consent was obtained from parents or legal guardians.

### Ethics

The present study was approved by the Ethical Committee for Research in Psychology of Università Cattolica del Sacro Cuore of Milan (approval number: pr87_24). Written informed consent was obtained from parents or legal guardians prior to children’s participation.

### Procedures

Data collection was conducted in collaboration with the recruited schools and followed a standardized administration protocol and was structured in two data collection phases conducted 5 to 7 days apart.

During the first phase, each child individually completed the Personal Interests Questionnaire for Children (PIQ-C), a brief open-ended self-report designed to collect information relevant for narrative personalization, administered individually in a quiet room within the school setting. The PIQ-C was adapted from the Personal Interests Questionnaire for adults (PIQ ^12^), originally developed to support individualized narrative feedback. To ensure developmental appropriateness for children aged 9 to 10 years, the questionnaire was translated into Italian and substantially revised. Revisions included reducing item length and linguistic complexity, removing content related to adult experiences (e.g., employment), and excluding prompts requiring abstract or highly reflective thinking that proved too demanding during pilot testing. Pilot testing also indicated that children had difficulty selecting single preferences; therefore, several items were reformulated to allow multiple responses. The final PIQ-C consists of eight open-ended items assessing identifying information, school preferences, admired figures, fears, perceived strengths, and physical self-description.

In the second phase, children were again individually invited to a quiet room were presented the fable corresponding to their assigned experimental condition, differing in the degree of personalization of the fable used as feedback material: (a) a non-personalized version, (b) a minimally personalized version including the child’s name and gender, (c) a moderately personalized version incorporating additional biographical elements (e.g., physical characteristics, a salient fear, and a personal strength), and (d) a fully personalized version that additionally reflected the child’s peer context and an admired helper figure.

All fables were developed according to theoretical principles of individualized feedback within the Therapeutic Assessment framework for children and shared the same narrative structure, plot, and moral message, differing only in the degree of personalization embedded in the text.

Immediately after reading the fable, participants completed post-test questionnaires assessing emotional engagement with the story and perceived narrative quality. Sessions lasted approximately 15 to 20 minutes and were conducted by trained researchers using a standardized script. No debriefing or interpretative discussion was provided to avoid influencing children’s spontaneous evaluations of the narrative material.

***Experimental condition: The Fable***

A fable was developed for each participant, with varying levels of personalization based on information provided in the PIQ-C. Each story featured a child protagonist characterized by specific personal strengths who was required to face a significant fear in a peer context to avoid shame or humiliation. During the narrative, the protagonist encountered an admired figure who encouraged the use of these personal qualities to cope with the challenging situation. The moral, conveyed in the final lines, emphasized that the solution adopted by the protagonist was shared with peers and met with curiosity and acceptance, highlighting that situations perceived as challenging are often common among children. The story concluded by conveying the message that facing difficult situations through self-expression, rather than excessive concern about others’ judgments, is generally the most adaptive response.

Four experimental versions of the fable were created, corresponding to four levels of personalization. All versions shared the same narrative structure, plot progression, and moral message, differing only in the degree and type of personalized elements embedded in the text. In the non-personalized condition, the story contained no individualized references, including gender. The minimally personalized version included the child’s name, gender, and age. The moderately personalized version additionally incorporated references to physical characteristics, a salient fear, and a personal strength identified in the PIQ-C. The fully personalized version further included explicit references to the child’s peer group and an admired helper figure identified by the child.

Children assigned to the non-personalized condition received a moderately personalized version of the fable after completion of data collection.

### Measures

***Fable Appreciation Questionnaire (FAQ)***

Children’s appreciation of the fable was assessed using the Fable Appreciation Questionnaire (FAQ), a post-test self-report measure specifically developed for this study to evaluate emotional engagement with the narrative and perceived formal qualities of the story.

The FAQ was derived from selected items adapted from two established therapeutic alliance measures. Two items were adapted from the Children’s Alliance Questionnaire (CAQ ^13^), and three items from the Vanderbilt Therapeutic Alliance Scale-Revised, Short Form (VTAS-R ^14^). Original items referring to the therapeutic relationship were conceptually reframed to assess the child’s relationship with the fable, replacing references to the therapist or mentor with references to the narrative. Item wording and syntax were simplified to ensure develpmental appropriateness for children aged 9 to 10.

Two additional items assessing formal aspects of the narrative (perceived readability and length) were included to capture potential structural differences across conditions. The final questionnaire consisted of seven items assessing overall liking of the story, desire to keep it, perceived emotional understanding and support, perceived usefulness of the solution presented, reading difficulty, and perceived length. Responses were provided on a 5-point Likert scale illustrated with emoticons.

Exploratory factor analysis supported a two-factor structure: Affective Engagement with the Fable (four items: e.g., “ *I like the fable I received*”; “ *I would like to keep the fable*”; 30% of variance; Cronbach’s α = .74) and Formal Qualities of the Fable (two items: “ *The fable was easy to read*”, “ *The fable was too long*”; 7% of variance; Cronbach’s α = .35).

## Analysis

Data analyses were conducted using IBM SPSS Statistics (version 31.0.0). To address the first research aim, independent-samples t tests were performed to compare children who received a non-personalized fable with those who received a personalized version (minimal, moderate, or full personalization) on affective engagement with the fable and perceived formal qualities (readability and length).

To address the second research aim, one-way analyses of variance (ANOVAs) were conducted to examine differences across the four personalization conditions (non-personalized, minimally personalized, moderately personalized, and fully personalized) on affective engagement and perceived formal qualities of the fables.

## Results

An independent-samples *t* test was conducted to compare children who received a non-personalized fable (n = 39) with those who received a personalized fable (n = 131).

Results indicated children in the personalized fable group showed higher affective engagement scores ( *Affective Engagement with the Fable* factor *;* M = 34.24, SD = 5.40) than those who read the non-personalized fable (M = 29.56, SD = 7.80; t(167) = -4.26, *p* < .001), with a moderate effect size ( *d* = 0.41).

Conversely, no significant group differences emerged for the perceived quality of the fable who received the non-personalized fable ( *Formal Qualities of the Fable* factor *; M* = 12.90, *SD* = 2.40) did not significantly differ from those who received a personalized fable ( *M* = 13.29, *SD* = 2.10, *t*(168) = - 0.998, *p* = .497).

A one-way analysis of variance (ANOVA) was conducted to examine differences in affective engagement with the fable across the four levels of personalization. The analysis revealed a statistically significant effect of personalization level (F(3) = 227.32; *p* < .001), with a moderate effect size (η ^2^ = .10).

Post hoc comparisons showed that children who received the non-personalized fable reported significantly lower levels of affective engagement (M = 29.56, SD = 7.80) compared with children who received a minimally personalized (M = 34.78, SD = 5.33; *p* = .004), moderately personalized (M = 34.78, SD = 4.70; *p* <.001), or fully personalized fable (M = 33.71, SD = 6.10; *p* = .013). Conversely, no significant differences emerged among the three personalized conditions ( Table 1).

**Table 1.** Affective engagement with the fable among personalization conditions.

Condition	M	SD	Non-personalized	Minimal personalization	Moderate personalization	Full personalization
Non-personalized	29.56	7.80	-	.004*	<.001**	.013
Minimal personalization	34.78	5.33		-	.968	.984
Moderate personalization	34.78	4.70			-	.841
Full personalization	33.71	6.10				-

Concerning perceived quality of the fable, no significant differences were found between the four personalization conditions (F(3)= .540; p =.655) ( Table 2).

**Table 2.** Perceived quality of the fable among personalization conditions.

Condition	M	SD	Non-personalized	Minimal personalization	Moderate personalization	Full personalization
Non-personalized	12.89	2.39	-	.978	.632	.836
Minimal personalization	13.09	2.02		-	.857	.971
Moderate personalization	13.46	1.65			-	.987
Full personalization	13.30	2.70				-

## Discussion

The present study examined whether different levels of personalization in a therapeutic fable influence children’s appreciation of the narrative, with a specific focus on affective engagement and perceived formal qualities. Overall, the findings support the hypothesis that personalization enhances children’s emotional involvement with the story, while having no substantial impact on perceived quality-related features.

Consistent with the first hypothesis, children who received a personalized fable reported significantly higher levels of affective engagement than those who received a non-personalized version. Exposure to any degree of personalization was associated with greater emotional involvement, perceived understanding, and perceived support conveyed by the story. These findings are consistent with previous research indicating that personalized narrative and feedback materials enhance emotional engagement by increasing personal relevance and fostering a sense of being recognized and mirrored. ^9, 11^

Importantly, even minimal personalization was sufficient to enhance affective engagement, with no significant differences among the minimally, moderately, and fully personalized conditions, suggesting a possible “threshold effect.” Once basic individualized elements - such as the child’s name or gender - were included, the story appeared to be experienced as personally relevant, and further personalization did not yield additional benefits.

In contrast, personalization did not influence children’s evaluations of the fable’s formal qualities, such as readability and length. This indicates that personalization selectively affects emotional and relational dimensions of children’s experience, rather than cognitive or structural aspects of the narrative. From a theoretical perspective, personalized fables may function as symbolic mirrors that allow children to recognize aspects of themselves within a psychologically safe narrative space, fostering a sense of being seen and understood.

### Limitations

Several limitations of the present study should be acknowledged. First, the sample was limited to children aged 9 to 10 years, which restricts the generalizability of the findings to other developmental stages. Future studies should examine whether similar effects of personalization emerge in younger children or early adolescents, for whom narrative comprehension, emotional engagement, and sensitivity to personalization may differ.

Second, the study was conducted in a non-clinical context. While this design allowed for a controlled investigation of the effects of personalization on narrative engagement, responses to different levels of personalization may be more pronounced in clinical settings, particularly when therapeutic stories are used to communicate assessment feedback or emotionally salient information. Research conducted in applied and clinical contexts is therefore needed to evaluate the ecological validity and clinical relevance of the present findings.

## Clinical implications

The present findings suggest that personalized fables can enhance children’s emotional engagement and sense of being understood, even when personalization is minimal. Clinically, personalized narratives offer a developmentally sensitive way to communicate complex or emotionally salient information to children. By fostering a sense of being seen and recognized, this approach may strengthen openness to feedback and the therapeutic relationship.

## Ethical approval statement

The study was approved by the Ethical Committee for Research in Psychology of Università Cattolica del Sacro Cuore, Milan, Italy (approval number: pr87_24). All procedures were conducted in accordance with the ethical standards of the institutional research committee and with the 1964 Helsinki declaration and its later amendments.

## Data Availability

• Repository name: OSF. Effects of Personalization in Children’s Fables. https://doi.org/10.17605/OSF.IO/NPEXD [97]. • The project contains the following underlying data: Data.sav (Anonymized participant-level data used for the statistical analyses reported in the article). • Repository name: OSF. Effects of Personalization in Children’s Fables. https://doi.org/10.17605/OSF.IO/NPEXD [97]. • This project contains the following extended data: Appendix.docx (Reporting full original versions in Italian of the questionnaires used in the study). Data are available under the terms of the Creative Commons Attribution 4.0 International (CC-BY 4.0) licence.
