# Supplementary material for: Effect of Personalization in Children's Fables
Source: F1000Res. 2026 Mar 19;15:415. [Version 1] doi: 10.12688/f1000research.177575.1 (PMC13550881; doi:10.12688/f1000research.177575.1)
Supplement: Peer review history

# Reviewer report: Reviewer response for version 1

**DOI:** 10.5256/f1000research.195814.r492547

**Published:** 2026-07-06

**Authors:** Cristina Mazza

**Affiliations:** 1 Sapienza University of Rome, Rome, Italy

This study examined whether different levels of personalization in therapeutic fables influence children’s appreciation of the narrative. Findings showed that even minimal personalization increased children’s affective engagement, whereas higher levels of personalization did not further improve children’s evaluation of the fable’s formal qualities.

- Please clarify what is meant by the expression “therapeutic stories.”
- In the sentence “However, it still remains unclear whether increasing degrees of personalization can produce incremental benefits,” please better explain what is meant by “incremental benefits.”
- In the sentence “Since reading comprehension represented an essential skill to read narrative material, inclusion criteria included the absence of known cognitive or linguistic impairments,” please specify whether the absence of cognitive or linguistic impairments was based on self-reported absence of diagnosis or was assessed through standardized instruments.
- Please provide further details on the pilot testing of the PIQ-C.
- In the sentence “Children assigned to the non-personalized condition received a moderately personalized version of the fable after completion of data collection,” please clarify what the neutral version consisted of and explain the meaning of this statement.
- In light of the discussion, please refer only to “qualities” in the hypotheses, specifying in parentheses which qualities are intended, and avoid using the expression “cognitive complexity.”
- Please provide more detailed statistical information on the factor analyses that were conducted.

Is the work clearly and accurately presented and does it cite the current literature?

Partly

If applicable, is the statistical analysis and its interpretation appropriate?

Partly

Are all the source data underlying the results available to ensure full reproducibility?

Yes

Is the study design appropriate and is the work technically sound?

Partly

Are the conclusions drawn adequately supported by the results?

Yes

Are sufficient details of methods and analysis provided to allow replication by others?

Partly

Reviewer Expertise:

Clinical Psychology, assessment and evaluation

I confirm that I have read this submission and believe that I have an appropriate level of expertise to confirm that it is of an acceptable scientific standard, however I have significant reservations, as outlined above.

# Reviewer report: Reviewer response for version 1

**DOI:** 10.5256/f1000research.195814.r492549

**Published:** 2026-06-19

**Authors:** Emily L. Winter, Maria Rotolo

**Affiliations:** 1 Clinical Psychology, Touro University, New York, New York, USA; 2 Touro University, New York City, New York, USA

Thank you for the opportunity to review “Effect of Personalization in Children's Fables” for consideration in F1000 Research. Please note that this review was co-completed by a psychology faculty member and a doctoral student in psychology. The present piece discusses the involvement of children in the testing/therapeutic processes. This topic is quite relevant and salient given the push in clinical and research for therapeutic assessment and client-centered approaches. Fables have been explored in the literature, and certainly, more research is needed to understand the mechanisms for how to approach the fables.

 Based on the review, we recommend the following:

 Introduction

- Add more details of what the therapeutic stories looked like in the introduction section
- Perhaps provide an example of what a fable might look like, either in the text or in an appendix
- Please add research questions in addition to the hypotheses
- More details about therapeutic assessment: a background of what this is, and the general principles
  - Maybe a figure of what TA encompasses could be assistive
  - Link to the TA website for people to learn more as a resource
- Additional citations and references needed in the literature review; this section feels under-cited and would benefit from further explanation

Method

- How did the researchers assess for known cognitive or linguistic impairments?
- Was child assent collected? If yes, please document, and if not, please add in this as a limitation and why this was not completed
- Tense feels off in this sentence: “The final PIQ-C consists of eight open-ended items assessing identifying information, school preferences, admired figures, fears, perceived strengths, and physical self-description.”
- What are the psychometrics for the PIQ-C, such as evidence of reliability and validity?
  - It stated that the fables were translated and substantially revised to meet developmental needs. It is important to note if this affects the evidence of validity/reliability of the testing instruments being used.
- Please provide samples of each degree of personalization in an appendix to more clearly illustrate these nuances
- Should the PIQ-C be added to the measures section? More details on how these data were used and any output from those results. Provide sample questions of what this measure looks like.
- What are the psychometrics for the FAQ such as evidence of reliability and validity?
- Provide example questions
- Please provide an example of the scale with the emoticons as a figure.
- Statistics need to be italicized: *SD, M, n, F*
- Please include in-text qualitative tables of the children’s responses to the fables and group responses by themes, if possible
- More descriptions of the children who voluntarily participated in the research, such as SES, any pre-existing diagnoses, cognitive abilities, race/ethnicity, and schooling experience
  - It also may be interesting to see a comparison of children from Northern and Central Italy by region and by gender, and also note why Southern Italy was not included in the study.
  - If any of these data were not collected, please add this to the limitations section

Discussion

- What does “mirrored” mean? Further exploration of this term
- There were three ethical approval statements in the document. Please condense.
- What are the best practices for administering the fables in clinical work? Please add this to the clinical implications
- Add a limitation about the lack of parents’ perspective
- What are the future directions for the work?

Is the work clearly and accurately presented and does it cite the current literature?

Partly

If applicable, is the statistical analysis and its interpretation appropriate?

Yes

Are all the source data underlying the results available to ensure full reproducibility?

Yes

Is the study design appropriate and is the work technically sound?

Yes

Are the conclusions drawn adequately supported by the results?

Yes

Are sufficient details of methods and analysis provided to allow replication by others?

Partly

Reviewer Expertise:

Cognitive assessment, developmental assessment, eating disorders

We confirm that we have read this submission and believe that we have an appropriate level of expertise to confirm that it is of an acceptable scientific standard, however we have significant reservations, as outlined above.

# Reviewer report: Reviewer response for version 1

**DOI:** 10.5256/f1000research.195814.r469221

**Published:** 2026-04-07

**Authors:** Alison Wilkinson-Smith

**Affiliations:** 1 Psychiatry, The University of Texas Southwestern Medical Center, Dallas, TX, USA

This brief report by Aschieri *et al. *provides a unique perspective on the use of therapeutic fables with children. As a frequent user of this technique myself, I am pleased to see efforts towards demonstrating its effectiveness. The ways in which typically developing children respond to therapeutic fables could represent a sort of minimum baseline that could inform how children who are vulnerable or struggling in some way might respond. The choice to specifically examine emotional engagement in contrast to a more general positive response is a thoughtful one which directly addresses the goal of therapeutic fables. Overall, my suggestions below are aimed at improving the ability of the reader to generalize these findings. I think this study has direct implications for clinical practice as well as for future research, so I'd like the applications to be even clearer. 

 I found I had the most questions regarding the description of the sample. Given that Therapeutic Assessment has found an international audience, more information about the participants could help the reader compare them to children in other locations. I am particularly curious about:

- Racial and ethnic makeup of the sample
- Socioeconomic diversity (or lack thereof)
- Implications of geographic distribution as described

It would be helpful for the authors to include this information or acknowledge its lack of availability as a limitation. I am also curious if the authors looked for differences in affective engagement in males vs. females, or in central vs. northern location, as potential confounds. 

 Regarding inclusion criteria, I would like to know:

- How was the absence of known cognitive or linguistic impairments assessed?
- Were any other learning, developmental, or psychological conditions screened for?

Again, I recommend that the authors include this information in a brief statement or acknowledge it in the limitations.

 My final suggestion is to make the fable templates included in the appendix a bit clearer. Perhaps use samples instead of templates or clarify what wording would be changed according to the parenthetical reference. I am also unclear why some parenthetical insertions are numbers only. I surmise that those numbers refer to the relevant item of the questionnaire, but these details are not altered in that version of the story. If so, perhaps they need not be included.

 I am very appreciative of the opportunity to provide feedback on this brief report. I hope my suggestions will make this manuscript even stronger. Reading this article caused me to reflect immediately on the implications for my own practice, and I hope other readers will be similarly inspired whether they currently use therapeutic fables or not. I look forward to additional research to extend these findings to other groups of children.

Is the work clearly and accurately presented and does it cite the current literature?

Yes

If applicable, is the statistical analysis and its interpretation appropriate?

Yes

Are all the source data underlying the results available to ensure full reproducibility?

Yes

Is the study design appropriate and is the work technically sound?

Yes

Are the conclusions drawn adequately supported by the results?

Yes

Are sufficient details of methods and analysis provided to allow replication by others?

Yes

Reviewer Expertise:

Therapeutic Assessment of children and families

I confirm that I have read this submission and believe that I have an appropriate level of expertise to confirm that it is of an acceptable scientific standard.
